# The association of levels of physical activity with metabolic syndrome in rural Australian adults

**DOI:** 10.1186/1471-2458-9-273

**Published:** 2009-07-31

**Authors:** Clare Vaughan, Adrian Schoo, Edward D Janus, Benjamin Philpot, Nathalie Davis-Lameloise, Sing Kai Lo, Tiina Laatikainen, Erkki Vartiainen, James A Dunbar

**Affiliations:** 1 Greater Green Triangle University Department of Rural Health, A partnership between Flinders and Deakin Universities, PO Box 423, Warrnambool, 3280, Victoria, Australia; 2 Department of Medicine, University of Melbourne, Western Hospital, Footscray, 3011, Victoria, Australia; 3 Faculty of Health, Medicine, Nursing and Behavioural Science, Deakin University, Burwood, 3125, Victoria, Australia; 4 Division of Welfare and Health Promotion, National Institute for Health and Welfare (THL), Mannerheimintie 166, 00300 Helsinki, Finland

## Abstract

**Background:**

Physical activity (PA) reduces risk factors related to metabolic syndrome. Rurality influences the way people incorporate physical activity into daily life. The aim of this study is to determine the association of PA level with metabolic syndrome in a rural Australian population. The influence of adiposity on these associations is also investigated.

**Methods:**

Three cross-sectional population health surveys were conducted in south-east Australia during 2004–2006 using a random population sample (n = 1563, participation rate 49%) aged 25–74 years. PA was assessed via a self-administered questionnaire, and components of the metabolic syndrome via anthropometric measurements taken by specially trained nurses and laboratory tests.

**Results:**

Approximately one-fifth of participants were inactive in leisure-time and over one-third had metabolic syndrome (men 39%, women 33%; p = 0.022). There was an inverse association between level of PA and metabolic syndrome (p < 0.001). Men who were inactive in leisure-time were more than twice as likely and women more than three times as likely to have metabolic syndrome compared with those having high PA. Body mass index (BMI) is a mediating factor in the association between level of PA and metabolic syndrome.

**Conclusion:**

Some PA is better than none if adults, particularly women, are to reduce their risk of metabolic syndrome and associated vascular diseases. Specialised interventions that take rurality into consideration are recommended for adults who are inactive.

## Background

Regular physical activity (PA) has a mild or moderate effect on numerous metabolic and cardiovascular risk factors that constitute or are related to the metabolic syndrome [1]. Metabolic syndrome includes a clustering of abnormal components: abdominal obesity, fasting hyperglycemia, hypertension and dyslipidaemia (high triglycerides, low high-density lipoprotein (HDL) cholesterol). By identifying people with metabolic syndrome, irrespective of the definition used [2,3], individuals who are at increased long-term risk of type 2 diabetes mellitus (T2DM) and cardiovascular disease (CVD) can be identified, and this provides opportunity for prevention through lifestyle intervention [4,5].

Lifestyle intervention to reduce risk of vascular diseases is mainly through diet and PA modification. A contemporary position on optimal levels of PA for health benefits promotes incidental activity in conjunction with 30 minutes of moderate-intensity activity on most days of the week which includes muscle strengthening activities [6]. The guidelines do not caution against sedentariness however the case is building for this [7]. When measuring PA levels, some studies measure total PA while others measure the domains of leisure-time, occupational or commuting PA separately. A previous investigation of the current study sample reported that few of the rural Australian adults met the level of PA needed for health benefits; most were moderately active in leisure-time; the majority of men engaged in high-level occupational PA (mainly employed in agriculture, forestry and fishing) with women reporting mostly low-level PA occupations (such as administration, management, education and other services); and approximately one-third of participants actively commuted to work [8]. It was hypothesised that with mechanisation of rural occupations, men perceive they are more active than in reality. It was also suggested that few people actively commute to work because the distances regularly travelled are beyond the 5–10 kilometres suited to active commuting [9] and infrastructure to safely separate active commuters from motorised vehicles is limited. Leisure-time physical activity (LTPA) is defined in this study as voluntary and purposeful activity, carried out to improve one or more components of physical fitness. LTPA is the preferred measure for this study because the categories include a comprehensive list of examples from sport and recreation, home duties and gardening as well as walking and cycling.

Strong evidence exists to show that obesity is a major risk factor for metabolic syndrome [10] and low levels of PA contribute to overweight and obesity [11]. There is increasing evidence from population studies describing an inverse association between level of PA and the prevalence of metabolic syndrome [12-21], however, to the best of our knowledge none of these studies are rural nor do they make clear the influence of overweight and obesity on the association. The aim of this study is to investigate the association of level of LTPA with metabolic syndrome in a random sample of adults from a rural Australian population. The influence of adiposity on the association is also investigated.

## Methods

### Participants

Three cross-sectional population health surveys were carried out in the Greater Green Triangle (GGT) region of south-east Australia between October 2004 and October 2006. The region is predominantly agricultural with multiple small towns of less than 1000 people and the two largest towns have 23,000 and 13,000 residents. Independent age and sex stratified random samples of people aged 25 to 74 years were drawn from the electoral roll. The total sample was 1563 participants with a 49% response rate.

### Data collection and measurements

The survey methodology closely followed the WHO MONICA protocol [22] and recommendations from the European Health Risk Monitoring Project [23]. The surveys were comprised of a self-administered questionnaire which included questions related to level of PA, plus a series of anthropometric measurements and laboratory tests to quantify cardiovascular and metabolic risk factors. The questionnaire, together with an invitation to attend a health check, was sent by mail to all selected participants and nurses specially trained in the survey procedures conducted the measurements; an explanation of these processes has been described elsewhere [24]. The protocols and procedures for measuring height, weight, waist circumference and blood pressure, for computing body mass index (BMI), and for venous blood sampling to determine fasting blood glucose and lipid profile are also described in detail elsewhere [24].

LTPA was categorised as 1) high: participation in activities to maintain fitness (running, jogging, gymnastics, swimming, ball-games or heavy gardening) or regular training for competitions several days a week; 2) low to moderate: walking, cycling or participating in another form of light exercise (fishing, home duties, light gardening) for at least four hours per week; and 3) inactive: reading, watching TV or other passive pursuits.

Metabolic syndrome was defined in this study according to the International Diabetes Federation (IDF) definition, which is suitable for all ethnic groups [2]. In addition, the definition of the Third Report of the National Cholesterol Education Program Expert Panel on Detection, Evaluation, and Treatment of High Blood Cholesterol in Adults, Adult Treatment Panel III (NCEP ATP III) was used for the analysis [3]. The definitions are:

a) IDF criteria specify central obesity with waist circumference ≥ 94 cm for men and ≥ 80 cm for women of Europid origin, plus two or more of the following: systolic blood pressure ≥ 130 mmHg or diastolic blood pressure ≥ 85 mmHg or antihypertensive medication; fasting plasma glucose ≥ 5.6 mmol/L or previously diagnosed type 2 diabetes; plasma HDL-cholesterol <1.03 mmol/L for men and <1.29 mmol/L for women; plasma triglycerides ≥ 1.7 mmol/L.

b) NCEP ATP III criteria includes three or more of the following: waist circumference ≥ 102 cm for males and ≥ 88 cm for females; systolic blood pressure ≥ 130 mmHg or diastolic blood pressure ≥ 85 mmHg or antihypertensive medication; fasting plasma glucose ≥ 5.6 mmol/L or on medication for high blood glucose; HDL cholesterol <1.03 mmol/L for males and <1.30 mmol/L for females; triglycerides ≥ 1.7 mmol/L.

Ethics approval was received from Flinders University Clinical Research Ethics Committee. Informed consent was received from participants when they attended the clinical testing component of the survey.

### Statistical analyses

Statistical analyses were undertaken using Stata version 10.1. Two-sided independent t-tests and Pearson chi-square tests were used to investigate gender differences in study population characteristics (Table 1), and the association between LTPA and metabolic syndrome (Table 2). For further analyses, data were weighted by the age and sex distribution of the survey regions according to the electoral roll.

**Table 1:** Characteristics of study population [n,%]

	Male		Female		P-value
n	721	46.8	818	53.2	
Age (mean, SD)	55.8	12.1	55.0	11.7	0.182
Education years (mean, SD)	11.2	2.9	11.4	2.7	0.169
Leisure-time PA level					<0.001
Inactive	143	19.8	123	15.0	
Low/moderate	451	62.6	602	73.6	
High	127	17.6	93	11.4	
Cardiovascular Disease	87	12.1	52	6.4	<0.001
Vascular risk factors					
Standard alcohol drinks per week (mean, SD)	10.8	14.2	3.8	5.8	<0.001
Current smoker	126	17.5	108	13.4	
Ex-smoker	274	38.1	215	26.7	
Non-smoker	319	44.4	483	59.9	
BMI (≥30 kg/m^2^)	202	30.3	255	35.0	0.062
LDL-cholesterol (>2.5 mmol/L or on lipid lowering medication)	592	89.8	618	84.8	0.005
Metabolic Syndrome (IDF)	250	39.0	232	33.0	0.022
Waist circumference (≤102 cm for males and ≤88 cm for females)	453	68.3	529	72.6	0.083
Hypertension (≥130/85 or on antihypertensive medication)	439	64.4	434	57.1	0.005
Fasting plasma glucose (≥5.6 mmol/L or on an antidiabetic medication)	215	33.3	144	20.4	<0.001
HDL-cholesterol (males <1.03 mmol/L; females <1.30 mmol/L)	154	23.6	126	17.9	0.009
Triglycerides (≥1.7 mmol/L)	224	35.5	200	29.0	0.012

Cardiovascular disease includes stroke, myocardial infarction, coronary bypass surgery or coronary angioplasty

**Table 2:** Prevalence of metabolic syndrome (IDF, NCEP ATP III) and leisure-time physical activity

	IDF ^#^		NCEP ATP III ^#^	
LTPA level	n	%	n	%
Inactive	112	48.9	100	43.7
Low/moderate	327	35.7	280	30.7
High	43	21.5	36	18.1
Overall	482	35.9	862	64.1

^# ^Level of leisure-time PA and metabolic syndrome (p < 0.001)

Linear regression was used to compare the means of BMI, waist circumference, systolic blood pressure (SBP), diastolic blood pressure (DBP), fasting plasma glucose, HDL-cholesterol, and triglycerides among participants in the three LTPA levels (Additional file 1). The mean differences between LTPA levels and 95% confidence intervals are presented for males and females separately.

Three models were constructed. Model one included the covariates: study area, age, education, smoking status and alcohol consumption. The second model included BMI in addition to the above covariates whereas the third model included waist circumference instead of BMI in addition to the above covariates listed.

For the logistic regression analyses, dichotomous variables were created using the IDF criteria of metabolic syndrome and its components, in addition to BMI (cut-off 30 kg/m^2^) and the NCEP ATP III criteria of metabolic syndrome (Additional file 2). Odds ratios and 95% confidence intervals are presented for the 3 models as described above.

## Result

This study included 1539 rural adults (men 47%, women 53%) aged 25–74 years with data available for LTPA (Table 1). The majority of participants reported a low to moderate level of LTPA (68%) and nearly one-fifth were inactive in leisure-time. Over one-third of participants had metabolic syndrome (men 39%, women 33%; p = 0.022; Table 1). The prevalence of hypertension and the concentrations of fasting plasma glucose, HDL-cholesterol and triglycerides based on the IDF cut-off points were worse in men than in women (Table 1). The prevalence of BMI and waist circumference above the cut-off points were not significantly different between men and women. There was an inverse association between level of LTPA and metabolic syndrome based on IDF and NCEP ATP III criteria (p < 0.001) (Table 2).

Among men and women, there was a significant association between the three levels of LTPA and waist circumference the inactive group had the highest waist circumference and the high LTPA group the lowest (Additional file 1, model 1). Similar associations were generally found for LTPA and BMI. After BMI adjustment (Additional file 1, model 2), the association remained for waist circumference in men and not women. After adjustment for waist circumference (Additional file 1, model 3), the association with BMI was no longer significant for any group.

In women, the other components of metabolic syndrome significantly associated with level of LTPA were fasting plasma glucose, HDL cholesterol and triglycerides (Additional file 1, model 1). After adjustment for the effect of BMI (Additional file 1, model 2), and waist circumference (Additional file 1, model 3), significant associations remained for fasting plasma glucose.

Men who were inactive in leisure-time were more than twice as likely and women nearly four times more likely to have metabolic syndrome based on IDF inclusion criteria, compared with those having high LTPA (Additional file 2, model 1). Similar four-fold increases in probability of having metabolic syndrome were found for women, when data were analysed using the NCEP ATP III definition (Additional file 2, model 1).

Men and women who were inactive in leisure-time were approximately four times more likely to be obese (BMI ≥ 30 kg/m^2^) compared with those having high LTPA (Additional file 2, model 1); similar results were found for central obesity (waist circumference based on IDF cut-offs). Men with low to moderate LTPA were more likely than inactive men to have the fasting plasma glucose and triglyceride components of metabolic syndrome above cut-off points (Additional file 2, models 2 and 3). In contrast, women who were inactive in leisure-time were over three times more likely to have fasting plasma glucose or triglycerides components of metabolic syndrome above the IDF cut-offs compared with those having high LTPA.

## Discussion

To our knowledge, this study is the first to describe the inverse association between LTPA and metabolic syndrome in a randomly selected rural adult population. Both men and women who were inactive in leisure-time were more likely to have metabolic syndrome compared with those who had high LTPA. This was a robust finding based on the IDF definition of metabolic syndrome. Using the NCEP ATP III definition, the significant association was found for women only. We consider the IDF definition of metabolic syndrome more appropriate since central obesity is a defined component and there are ethnicity specific values. By contrast, the NCEP ATP III definition, for example, could include thin Asian people with hypertension and dyslipidaemia along with obese individuals [25].

The inverse association between LTPA and metabolic syndrome has previously been reported [12-21], however those studies either used the NCEP ATP III definition of metabolic syndrome, or were conducted in selected populations. Even though a range of instruments are used to measure PA, the relationships with metabolic syndrome have nevertheless been consistent. Currently, there is no gold standard for self-reported physical activity measurement [26]. The tool used in this study is a simple self-reported measure suited to the self-administered questionnaire format, and it has been used and reported elsewhere [27].

The reported findings on the influence of rurality on metabolic syndrome and its key component obesity are inconsistent [28-31]. As part of the phenomenon of epidemiological transition, developing countries tend to show urban obesity, and developed countries tend to show rural obesity. In Australia, there is some suggestion that rural and urban areas have a similar prevalence of obesity [24].

Several studies, one of which was conducted in a 'real-world' setting in rural Australia, have highlighted the importance of weight loss, particularly through reduced waist circumference as a primary target for prevention of T2DM [32-34]. It is of concern that one-fifth of participants in this study were inactive in leisure-time and that this group was most likely to be obese and have waist circumferences exceeding the IDF cut-off points. Our results show an association between obesity (BMI and waist circumference) and LTPA which is consistent with another Finnish population study that used the same tool as our study to measure LTPA [27]. To determine whether BMI and waist circumference were confounding factors in the relationship between LTPA and metabolic syndrome, we adjusted for the effect of these variables (Additional file 2). Our results suggest that BMI and waist circumference are mediating factors in the relationship, particularly for women and weight loss is likely to reduce risk of metabolic syndrome and therefore T2DM and CVD.

One of the strengths of the study is the random sample drawn from a rural adult population. Although the participation rate was modest (49%), a comparison of socioeconomic characteristics of survey participants with population statistics available indicated that participants closely represented the true populations of the areas [35]. Another strength is that a close comparison could be made with the study by Barengo et al. [27] since it used the same LTPA measure and similar methods of analysis. The present study was enhanced by the investigation of the relationships between LTPA and metabolic syndrome and its components.

A limitation of this study is that it relies on self-reported LTPA. Self-reported physical activity measures are associated with over-reporting [26] however such measures are widely used as indicators of activity, and the costs of more objective physical measures were beyond the scope of this study. Dietary habits were also self-reported in the survey; however there was no overall measure to include as a covariate in the analyses. Another limitation is the disparities found in men for fasting plasma glucose and triglycerides which remain unexplained. The result of no effect was also found in another Australian study, where the positive association between sedentary behaviour and fasting plasma glucose or triglyceride was found for women and not men [36].

The greatest public health gains from physical activity promotion come from increasing the physical activity level of people who are sedentary [11]. It has been suggested that in rural Australia, facilities and opportunities for LTPA and active commuting tend to be limited compared with urban areas as a result of smaller and more dispersed populations. Unstructured or low-intensity activity such as walking has been shown to reduce the risk of developing metabolic syndrome [37], and tai chi has shown promise [38]. These activities do not require specialist facilities and are suited to a low intensity start-point with gradual progressions making them appropriate for inactive adults living in rural areas. Active obese individuals have lower cardiovascular disease risk than those who are sedentary and obese [39], and the types of activities suggested above are also appropriate for rural obese people. The challenge for health professionals is how to best intervene with this difficult to reach group.

Health professionals working in rural areas require specialised skills to support people who may not perceive themselves as inactive, to plan for, and engage in LTPA. Rural health professionals require an understanding of occupational physical activity in the rural context as well as the challenges of active commuting in these regions and the limited opportunities and facilities for LTPA. In 2006, a project conducted in the same region of rural Australia as the current study, reported on an effective group lifestyle modification program designed for people at high risk of developing T2DM [33]. More women than men chose to participate in the program and the results of this study suggest that women are particularly vulnerable to cardiovascular disease if they are inactive. Program success was attributed to factors that include the theory based health behaviour principles that underpinned the program and the fact that local health professionals who delivered the program were trained in non-directive counselling techniques and group facilitation. The group program mentioned above supports these processes and the group helped to generate possible solutions that individuals could adopt for themselves. The program is particularly suited to increase LTPA in inactive adults with metabolic syndrome who live in rural areas.

## Conclusion

At least 20 percent of rural Australian adults in this study were physically inactive in their leisure-time and this inactivity was associated with an increased probability of having metabolic syndrome, particularly in women. Some physical activity is better than none if adults are to reduce their risk of metabolic syndrome and other vascular diseases.

## Competing interests

The authors declare that they have no competing interests.

## Authors' contributions

CV coordinated writing the manuscript. AS initiated the article and contributed to writing the manuscript. EDJ initiated, organised and supervised the surveys, contributed to data interpretation and writing the manuscript. BP conducted the statistical analysis and contributed to writing the manuscript. NDL contributed to writing the manuscript. SKL provided expert guidance in manuscript planning and statistical analysis. TL initiated, organised and supervised the surveys, contributed to data interpretation and writing the manuscript. EV was involved in the survey design and contributed to data analysis. JAD was the lead investigator in the three surveys, is the guarantor and contributed to writing the manuscript. All authors read and approved the final version of the manuscript.

## Pre-publication history

The pre-publication history for this paper can be accessed here:



## Supplementary Material

Additional file 1 **Supplemental Table**. Mean differences in cardiovascular and metabolic risk factors according to level of leisure-time PA among rural Australian adult Click here for file

Additional file 2 **Supplemental table**. Indicators of elevated cardiovascular and metabolic risk factors according to leisure-time PA level among adults in a rural Australian population Click here for file
