# Supplementary material for: The association of levels of physical activity with metabolic syndrome in rural Australian adults
Source: BMC Public Health. 2009 Jul 31;9:273. doi: 10.1186/1471-2458-9-273 (PMC2736941; doi:10.1186/1471-2458-9-273)
Supplement: Additional file 1 — Supplemental Table. Mean differences in cardiovascular and metabolic risk factors according to level of leisure-time PA among rural Australian adult [file 1471-2458-9-273-S1.doc]

## Additional file 1: Mean differences in cardiovascular and metabolic risk factors according to level of leisure-time PA among rural Australian adults

|  | BMI  (kg/m2) | | Waist circumference  (cm) | | SBP  (mmHg)^a^ | | DBP  (mmHg)^a^ | | Fasting Plasma Glucose^b^  (mmol/L) | | HDL-Cholesterol  (mmol/L) | | Triglycerides  (mmol/L) | |
| --- | --- | --- | --- | --- | --- | --- | --- | --- | --- | --- | --- | --- | --- | --- |
|
| Men (n) | 654 | | 650 | | 495 | | 495 | | 593 | | 639 | | 604 | |
| Model 1 | Mean | (95% CI) | Mean | (95% CI) | Mean | (95% CI) | Mean | (95% CI) | Mean | (95% CI) | Mean | (95% CI) | Mean | (95% CI) |
| Low/Mod - Inactive | -0.95 | (-2.23,0.34) | -3.39^*^ | (-6.72,-0.05) | -0.81 | (-5.00,3.37) | -1.73 | (-4.68,1.21) | -0.02 | (-0.15,0.11) | 0.05 | (-0.03,0.13) | -0.12 | (-0.50,0.26) |
| High - Inactive | -2.03^*^ | (-3.46,-0.61) | -7.58^*^ | (-11.19,-3.98) | -0.68 | (-5.27,3.91) | -3.27 | (-6.68,0.14) | 0.00 | (-0.15,0.15) | 0.08 | (-0.03,0.19) | -0.20 | (-0.63,0.23) |
| High - Low/Mod | -1.09^*^ | (-2.09,-0.08) | -4.20^*^ | (-6.74,-1.65) | 0.13 | (-3.23,3.49) | -1.54 | (-4.17,1.09) | 0.02 | (-0.09,0.14) | 0.03 | (-0.06,0.11) | -0.08 | (-0.31,0.15) |
| Model 2 |  |  |  |  |  |  |  |  |  |  |  |  |  |  |
| Low/Mod - Inactive | - | - | -1.27 | (-2.58,0.04) | -0.26 | (-4.39,3.87) | -1.41 | (-4.38,1.55) | 0.00 | (-0.13,0.13) | 0.03 | (-0.05,0.10) | -0.06 | (-0.43,0.31) |
| High - Inactive | - | - | -2.81^*^ | (-4.39,-1.22) | 0.23 | (-4.27,4.73) | -2.78 | (-6.29,0.72) | 0.04 | (-0.11,0.18) | 0.03 | (-0.06,0.13) | -0.09 | (-0.51,0.32) |
| High - Low/Mod | - | - | -1.54^*^ | (-2.70,-0.38) | 0.49 | (-2.78,3.75) | -1.37 | (-3.95,1.21) | 0.04 | (-0.08,0.15) | 0.00 | (-0.07,0.08) | -0.04 | (-0.26,0.19) |
| Model 3 |  |  |  |  |  |  |  |  |  |  |  |  |  |  |
| Low/Mod - Inactive | 0.26 | (-0.24,0.77) | - | - | 0.17 | (-3.99,4.34) | -1.14 | (-4.11,1.83) | 0.00 | (-0.13,0.13) | 0.02 | (-0.06,0.09) | -0.04 | (-0.41,0.33) |
| High - Inactive | 0.57 | (-0.06,1.20) | - | - | 0.93 | (-3.68,5.55) | -2.39 | (-5.96,1.18) | 0.05 | (-0.10,0.20) | 0.01 | (-0.09,0.11) | -0.05 | (-0.46,0.36) |
| High - Low/Mod | 0.31 | (-0.15,0.76) | - | - | 0.76 | (-2.54,4.06) | -1.25 | (-3.88,1.38) | 0.05 | (-0.07,0.17) | -0.01 | (-0.09,0.07) | -0.01 | (-0.24,0.22) |
|  |  |  |  |  |  |  |  |  |  |  |  |  |  |  |
| Women (n) | 701 | | 701 | | 509 | | 509 | | 640 | | 677 | | 655 | |
| Model 1 | Mean | (95% CI) | Mean | (95% CI) | Mean | (95% CI) | Mean | (95% CI) | Mean | (95% CI) | Mean | (95% CI) | Mean | (95% CI) |
| Low/Mod - Inactive | -3.21^*^ | (-5.27,-1.15) | -6.62^*^ | (-10.52,-2.71) | -2.63 | (-6.67,1.41) | -1.53 | (-4.37,1.31) | -0.22^*^ | (-0.37,-0.07) | 0.10^*^ | (0.02,0.19) | -0.24^*^ | (-0.43,0.06) |
| High - Inactive | -5.21^*^ | (-7.47,-2.95) | -11.37^*^ | (-15.97,-6.77) | -3.77 | (-9.53,1.99) | -1.16 | (-5.12,2.80) | -0.28^*^ | (-0.45,-0.11) | 0.12^*^ | (0.00,0.23) | -0.37^*^ | (-0.61,0.13) |
| High - Low/Mod | -2.00^*^ | (-3.24,-0.77) | -4.75^*^ | (-7.95,-1.55) | -1.14 | (-5.90,3.62) | 0.37 | (-2.78,3.53) | -0.06 | (-0.18,0.05) | 0.01 | (-0.09,0.11) | -0.13 | (-0.28,0.03) |
| Model 2 |  |  |  |  |  |  |  |  |  |  |  |  |  |  |
| Low/Mod - Inactive | - | - | -0.08 | (-2.24,2.09) | -0.83 | (-4.88,3.22) | -0.37 | (-3.40,2.67) | -0.15^*^ | (-0.29,0.00) | 0.03 | (-0.05,0.11) | -0.13 | (-0.29,0.03) |
| High - Inactive | - | - | -0.64 | (-3.38,2.11) | -0.61 | (-6.41,5.18) | 0.78 | (-3.33,4.88) | -0.17^*^ | (-0.34,0.00) | 0.00 | (-0.11,0.12) | -0.19 | (-0.40,0.03) |
| High - Low/Mod | - | - | -0.56 | (-2.38,1.26) | 0.22 | (-4.44,4.87) | 1.14 | (-1.97,4.26) | -0.02 | (-0.14,0.10) | -0.03 | (-0.12,0.07) | -0.06 | (-0.21,0.10) |
| Model 3 |  |  |  |  |  |  |  |  |  |  |  |  |  |  |
| Low/Mod - Inactive | -0.69 | (-1.76,0.38) | - | - | -1.05 | (-5.12,3.02) | -0.29 | (-3.16,2.57) | -0.15^*^ | (-0.30, 0.00) | 0.03 | (-0.06,0.12) | -0.12 | (-0.29,0.05) |
| High - Inactive | -0.94 | (-2.20,0.32) | - | - | -1.02 | (-6.93,4.88) | 0.74 | (-3.25,4.74) | -0.17 | (-0.34, 0.00) | 0.00 | (-0.12,0.11) | -0.17 | (-0.40,0.06) |
| High - Low/Mod | -0.25 | (-0.97,0.48) | - | - | 0.03 | (-4.74,4.80) | 1.04 | (-2.05,4.13) | -0.02 | (-0.14, 0.10) | -0.03 | (-0.13,0.07) | -0.05 | (-0.21,0.10) |
| Legend: BMI, Body mass index; CI, confidence interval; DBP, diastolic blood pressure; SBP, systolic blood pressure, Mod, moderate.  Model 1: Adjusted for age, area, education, smoking, and alcohol intake; Model 2: Adjusted for BMI, age, area, education, smoking, and alcohol intake; Model 3: Adjusted for waist circumference, age, area, education, smoking, and alcohol intake.  ^a^ excludes participants on antihypertensive medication; ^b^ excludes participants on diabetes medication; *p-value <0.05 | | | | | | | | | | | | | | |
