# Supplementary material for: The association of levels of physical activity with metabolic syndrome in rural Australian adults
Source: BMC Public Health. 2009 Jul 31;9:273. doi: 10.1186/1471-2458-9-273 (PMC2736941; doi:10.1186/1471-2458-9-273)
Supplement: Additional file 2 — Supplemental table. Indicators of elevated cardiovascular and metabolic risk factors according to leisure-time PA level among adults in a rural Australian population [file 1471-2458-9-273-S2.doc]

## Additional file 2 - Indicators of elevated cardiovascular and metabolic risk factors according to leisure-time PA among rural Australian adults

|  | BMI  (>=30kg/m2) | | Waist circumference  (IDF) | | Blood Pressure  (IDF) | | Fasting Plasma Glucose  (IDF) | | HDL-Cholesterol  (IDF) | | Triglycerides  (IDF) | | Metabolic  Syndrome  (IDF) | | Metabolic  Syndrome  (NCEP ATP III) | |
| --- | --- | --- | --- | --- | --- | --- | --- | --- | --- | --- | --- | --- | --- | --- | --- | --- |
|
| Men (n) | 654 | | 650 | | 667 | | 632 | | 639 | | 618 | | 628 | | 629 | |
| Model 1 | OR | (95% CI) | OR | (95% CI) | OR | (95% CI) | OR | (95% CI) | OR | (95% CI) | OR | (95% CI) | OR | (95% CI) | OR | (95% CI) |
| Inactive | 1 |  | 1 |  | 1 |  | 1 |  | 1 |  | 1 |  | 1 |  | 1 |  |
| Low / Moderate | 0.59 | (0.35-1.01) | 0.61 | (0.33-1.14) | 0.93 | (0.54-1.60) | 1.50 | (0.90-2.50) | 0.84 | (0.46-1.52) | 1.47 | (0.86-2.50) | 0.78 | (0.46-1.33) | 0.94 | (0.57-1.57) |
| High | 0.26^*^ | (0.13-0.54) | 0.29^*^ | (0.14-0.59) | 0.94 | (0.47-1.88) | 1.35 | (0.70-2.62) | 0.59 | (0.26-1.31) | 1.04 | (0.52-2.08) | 0.41^*^ | (0.21-0.82) | 0.56 | (0.28-1.11) |
| Model 2 |  |  |  |  |  |  |  |  |  |  |  |  |  |  |  |  |
| Inactive |  |  | 1 |  | 1 |  | 1 |  | 1 |  | 1 |  | 1 |  | 1 |  |
| Low / Moderate | - | - | 0.51 | (0.24-1.10) | 1.00 | (0.56-1.81) | 1.67^*^ | (1.00-2.78) | 0.97 | (0.51-1.85) | 1.80^*^ | (1.02-3.16) | 1.02 | (0.51-2.03) | 1.35 | (0.68-2.69) |
| High | - | - | 0.35^*^ | (0.13-0.96) | 1.10 | (0.52-2.34) | 1.64 | (0.83-3.24) | 0.75 | (0.32-1.74) | 1.37 | (0.67-2.83) | 0.67 | (0.26-1.41) | 0.92 | (0.37-2.29) |
| Model 3 |  |  |  |  |  |  |  |  |  |  |  |  |  |  |  |  |
| Inactive | 1 |  |  |  | 1 |  | 1 |  | 1 |  | 1 |  | 1 |  | 1 |  |
| Low / Moderate | 0.90 | (0.32-2.58) | - | - | 1.06 | (0.58-1.94) | 1.71^*^ | (1.03-2.83) | 0.98 | (0.51-1.88) | 1.82^*^ | (1.03-3.20) | 1.11 | (0.54-2.26) | 1.47 | (0.75-2.87) |
| High | 0.69 | (0.18-2.69) | - | - | 1.21 | (0.56-2.60) | 1.74 | (0.89-3.43) | 0.80 | (0.35-1.86) | 1.45 | (0.70-2.99) | 0.78 | (0.33-1.87) | 1.19 | (0.48-2.92) |
|  |  |  |  |  |  |  |  |  |  |  |  |  |  |  |  |  |
| Women (n) | 701 | | 701 | | 730 | | 679 | | 677 | | 663 | | 677 | | 671 | |
| Model 1 |  |  |  |  |  |  |  |  |  |  |  |  |  |  |  |  |
| Inactive | 1 |  | 1 |  | 1 |  | 1 |  | 1 |  | 1 |  | 1 |  | 1 |  |
| Low / Moderate | 0.47^*^ | (0.27-0.82) | 0.61 | (0.29-1.29) | 0.89 | (0.51-1.55) | 0.66 | (0.38-1.15) | 0.72 | (0.38-1.38) | 0.42^*^ | (0.24-0.73) | 0.50^*^ | (0.28-0.88) | 0.44^*^ | (0.24-0.79) |
| High | 0.23^*^ | (0.10-0.54) | 0.28^*^ | (0.11-0.68) | 0.82 | (0.35-1.92) | 0.29^*^ | (0.10-0.85) | 0.57 | (0.20-1.65) | 0.26^*^ | (0.11-0.63) | 0.28^*^ | (0.10-0.82) | 0.25^*^ | (0.08-0.76) |
| Model 2 |  |  |  |  |  |  |  |  |  |  |  |  |  |  |  |  |
| Inactive |  |  | 1 |  | 1 |  | 1 |  | 1 |  | 1 |  | 1 |  | 1 |  |
| Low / Moderate | - | - | 1.06 | (0.41-2.72) | 1.38 | (0.77-2.48) | 0.98 | (0.54-1.77) | 1.027 | (0.51-2.08) | 0.55^*^ | (0.32-0.97) | 0.79 | (0.41-1.54) | 0.72 | (0.35-1.49) |
| High | - | - | 0.74 | (0.22-2.51) | 1.39 | (0.56-3.43) | 0.58 | (0.18-1.79) | 1.05 | (0.35-3.16) | 0.44 | (0.18-1.06) | 0.66 | (0.21-2.12) | 0.66 | (0.20-2.23) |
| Model 3 |  |  |  |  |  |  |  |  |  |  |  |  |  |  |  |  |
| Inactive | 1 |  |  |  | 1 |  | 1 |  | 1 |  | 1 |  | 1 |  | 1 |  |
| Low / Moderate | 0.65 | (0.27-1.56) | - | - | 1.37 | (0.78-2.43) | 1.04 | (0.57-1.90) | 1.07 | (0.51-2.24) | 0.56^*^ | (0.32-0.98) | 0.81 | (0.39-1.69) | 0.72 | (0.31-1.66) |
| High | 0.60 | (0.17-2.14) | - | - | 1.30 | (0.53-3.20) | 0.62 | (0.18-2.09) | 1.15 | (0.36-3.67) | 0.47 | (0.19-1.17) | 0.72 | (0.20-2.55) | 0.72 | (0.19-2.78) |
| Legend: BMI, Body mass index; CI, confidence interval; DBP, diastolic blood pressure; OR, odds ratio; SBP, systolic blood pressure. Model 1: Adjusted for age, area, education, smoking, and alcohol intake.  Model 2: Adjusted for BMI, age, area, education, smoking, and alcohol intake. Model 3: Adjusted for waist circumference, age, area, education, smoking, and alcohol intake; *p-value <0.05 | | | | | | | | | | | | | | | | |
